# Assessment of Motivations, Treatment Risks, and Oral Health in Adults with Fixed Orthodontic Care: A Cross-Sectional Study

**DOI:** 10.3390/medicina60071149

**Published:** 2024-07-17

**Authors:** Antonija Tadin, Marija Badrov, Branimira Mikelic Vitasovic, Lidia Gavic

**Affiliations:** 1 Department of Restorative Dental Medicine and Endodontics, Study of Dental Medicine, University of Split School of Medicine, 21000 Split, Croatia; atadin@mefst.hr (A.T.); mb91802@mefst.hr (M.B.); 2 Department of Maxillofacial Surgery, Clinical Hospital Centre Split, 21000 Split, Croatia; 3 Private Dental Clinic, 21000 Split, Croatia; bmikelic@yahoo.com

**Keywords:** health behavior, factors influencing orthodontic treatment, oral health, oral hygiene

## Abstract

*Aim:* This cross-sectional study aims to investigate the reasons why adults seek orthodontic treatment, their perceived treatment risks, oral hygiene habits, and awareness of oral health. *Materials and Methods:* This study, which involved 246 adults, used a self-report questionnaire to collect sociodemographic data and examine the participants’ understanding of oral health, self-assessment of oral health status, adherence to oral hygiene routines, and use of oral hygiene products. In addition, the reasons for opting for orthodontic treatment, negative associations, and expected outcomes were examined. The collected data were subjected to statistical analysis, including descriptive and regression methods. *Results:* The results showed that factors such as previous oral health education and regular use of specialized toothbrushes, including rotary toothbrushes, were significantly associated with higher knowledge levels (*p* < 0.05). However, daily oral hygiene practices were suboptimal, with the low utilization of aids: only 58.9% reported using interdental brushes, and 36.6% used dental floss regularly. Commonly reported dental problems included food debris between teeth (46.3%), tartar (35.4%), and tooth sensitivity to cold (26.4%). The primary motivations for orthodontic treatment were aesthetic improvement (63.8%), enhancements in oral function (49.6%), and addressing psychosocial concerns (42.7%). In contrast, the most frequently mentioned negative aspects associated with fixed orthodontic treatment were aesthetic concerns (30.9%), treatment duration (27.6%), and mouth ulcers (24.0%). *Conclusions:* Based on the research findings, regular oral hygiene education is recommended for adult patients considering fixed orthodontic therapy, including the use of additional tools such as interdental brushes and floss to improve oral health and reduce the risk of complications during treatment. The importance of chemical compounds in dentifrices and mouthwashes cannot be overstated, as they play a crucial role in addressing various dental issues. In addition, it is crucial to carefully weigh the pros and cons of therapy and to visit a dentist regularly to maintain oral health and monitor progress during treatment.

## 1. Introduction

Orthodontic therapy not only aligns teeth but also enhances overall oral health. Adults pursue these treatments for a multitude of reasons, influenced by both professional and social factors. Among those undergoing fixed orthodontic treatment, expectations typically include heightened self-confidence, enhanced oral health, and improvements in both aesthetics and functionality. These anticipated benefits underscore the comprehensive impact that orthodontic therapy can have on patients’ lives [1,2,3].

Patients considering fixed orthodontic treatment should be prepared for potential challenges [4]. Discomfort can take the form of pain and sensitivity of the teeth and gums, especially after braces or adjustments have been placed. The duration of treatment, which usually extends over several months to years, can also be a problem for some patients. Factors that contribute to orthodontic patients’ discomfort include the type of appliance used, the amount of force applied during the initial phase of treatment, previous experiences of pain and various emotional, cognitive, and environmental factors such as culture, gender, and age. In addition, patients may face dietary restrictions, oral hygiene, speech disorders, and social interactions during treatment [5,6,7,8]. Understanding these issues is crucial for improving dental health practices in Croatia, potentially leading to nationwide improvements in oral hygiene and treatment outcomes. This study provides valuable insights into how orthodontic therapy influences Croatian dental health practices.

Maintaining oral hygiene in patients undergoing fixed orthodontic treatment can be challenging due to the presence of brackets, wires, and other orthodontic appliances that can interfere with proper tooth cleaning. However, regular and thorough oral hygiene is crucial to prevent plaque increase, tooth decay, and gum inflammation. Orthodontists typically educate their patients through both verbal instructions and written materials on the importance of oral hygiene and the proper use of auxiliary aids, particularly specialized orthodontic toothbrushes, interdental brushes, and dental floss. These tools are essential for cleaning between teeth and around brackets to maintain optimal oral health during orthodontic treatment. Apart from mechanical cleaning, there are various chemical compounds in dentifrices and mouthwashes that address common dental issues during orthodontic therapy, such as tartar buildup, sensitivity, bad breath, mouth ulcers, plaque, and periodontal disease [9,10,11,12]. For tartar control, pyrophosphates prevent tartar formation by binding calcium and magnesium ions, while zinc citrate disrupts tartar formation and has antibacterial properties. To manage dentin sensitivity, potassium nitrate desensitizes nerves, and strontium chloride blocks tubules in dentin to reduce sensitivity. For bad breath, chlorhexidine is a powerful antimicrobial, cetylpyridinium chloride (CPC) reduces plaque and kills odor-causing bacteria, and essential oils like eucalyptol, menthol, and thymol provide antibacterial effects and mask bad breath. To treat mouth ulcers, benzocaine provides pain relief, hydrogen peroxide cleans ulcers and reduces bacterial load, and aloe vera soothes and heals. For antiplaque benefits, triclosan inhibits bacterial growth, and fluorides reduce bacterial growth and prevent cavities. For periodontal disease, chlorhexidine reduces gingivitis and plaque, essential oils reduce plaque and gingivitis, and stannous fluoride reduces inflammation and bacterial load [13].

Despite regular oral hygiene practices and education efforts, patients with fixed orthodontic appliances often experience various oral issues such as cavities, plaque accumulation, gum inflammation, and halitosis. These challenges arise due to the presence of harder-to-reach areas for cleaning around brackets and wires, which can trap food particles and bacteria, leading to increased susceptibility to dental problems [14,15,16,17]. Therefore, patients should receive detailed oral hygiene instructions from their orthodontist regularly during treatment, along with biannual dental check-ups [16,17].

Numerous studies have been conducted worldwide to assess the oral health knowledge, oral health status, and oral hygiene practices of orthodontic patients. Although many studies have been conducted on oral health knowledge and the implementation of preventive measures in children and adolescents [9,12], such studies are rarer in the adult population [11,18]. To date, few studies have been conducted in Croatia on orthodontic patients to investigate the factors influencing their decision to undergo treatment, the unpleasant features associated with orthodontic treatment, and the evaluation of oral health and oral hygiene practices in these patients [11,19,20]. Thus, this study aims to investigate several key aspects among adult patients undergoing fixed orthodontic therapy. Specifically, it seeks to evaluate self-assessed oral health and oral hygiene practices, and to assess their knowledge of oral health. Additionally, this study aims to identify the factors influencing patients’ motivation and reasons for choosing orthodontic treatment. Furthermore, it examines the negative characteristics associated with fixed orthodontic therapy and the patients’ expectations from the treatment. By exploring these dimensions, this research provides a comprehensive understanding of the experiences and perceptions of adult orthodontic patients.

## 2. Materials and Methods

### 2.1. Study Design and Population

This cross-sectional study was conducted from April 2023 to April 2024 in a private dental clinic in Split, Croatia, using a convenience sample. Data collection was conducted by means of a survey for which ethical approval was granted by the Ethics Committee of the School of Medicine, University of Split, Croatia, to ensure compliance with established guidelines, including the World Medical Association’s Declaration of Helsinki. Participation was voluntary and anonymity of respondents was maintained. The inclusion criteria consisted of patients aged 18 years and older who had undergone fixed orthodontic treatment for at least three months to assess the impact of the appliance on their quality of life. Exclusion criteria included adolescents, people with mental or physical disabilities, chronically ill patients, pregnant women, patients with dentofacial deformities such as cleft palate, patients undergoing or having undergone treatment with a non-fixed appliance, and patients who did not answer the questionnaire.

During the study period, all adult patients attending a private dental clinic in Split, Croatia (N = 264), who met the inclusion criteria, were invited to complete an anonymous self-administered questionnaire during their dental appointment. The aim of this study was explained to participants prior to questionnaire completion. A total of 246 participants completed the questionnaire, resulting in a response rate of 93.1%. The questionnaire was not completed by adult patients who exhibited any of the exclusion criteria.

### 2.2. Questionnaire

This study’s data were collected using a questionnaire derived from various surveys, all sharing a consistent thematic focus [9,12,21,22,23,24,25,26,27,28,29]. Before the main survey, a pilot survey was conducted with 15 participants to assess the validity and reliability of the questionnaire. Pilot testing was conducted online with respondents who were not part of the primary study and were not patients of the practice where the research took place. Respondents willingly participated, and the survey completion time was approximately 10 min. Based on the feedback and results from the pilot survey, adjustments were made to improve the clarity and comprehensibility of the questionnaire items, ensuring their reliability and accuracy for the main study. The Cronbach’s alpha coefficient of internal consistency for this questionnaire was tested on pilot study participants, resulting in a value of 0.617, indicating acceptable reliability.

The questionnaire, consisting of 86 questions divided into eight sections, began with the first section (Q1–Q5) collecting sociodemographic data from respondents, including gender, age, education level, employment status, and socioeconomic status. The second section (Q6–Q15) focused on assessing oral health knowledge, with respondents choosing from three options: “Yes”, “No”, or “I don’t know”. A scoring system was used in this study where a correct answer (“Yes”) received a score of one, and an incorrect answer received a score of zero. The total score for each respondent was calculated based on the number of correct answers, providing a quantitative measure of their knowledge level. The third section (Q16–Q25) included questions related to the self-assessment of personal oral health and hygiene, knowledge about oral health and hygiene, sources of oral health information, attitudes toward the importance of oral health, and the duration of orthodontic therapy and its interference in maintaining oral hygiene. The fourth section (Q26–Q36) detailed self-reported oral issues experienced by respondents, covering aspects such as plaque, dental calculus, food debris, bleeding gums, and dentin hypersensitivity. The fifth section (Q37–Q48) comprised questions concerning oral hygiene practices. These included inquiries about the frequency of using oral hygiene aids, preferred toothbrush hardness, intervals for replacing a toothbrush, and the type of toothbrush used. The sixth section (Q49–Q58) comprised 10 questions addressing various factors influencing orthodontic treatment, including aesthetics, teeth straightening, and enhancing oral function. The seventh section (Q59–Q77) included 19 questions about negative characteristics associated with orthodontic treatments, such as pain, duration of therapy, dietary issues, and oral hygiene maintenance. The final section (Q78–Q86) consisted of questions regarding expectations from orthodontic treatment.

### 2.3. Data Analysis

To assess the normality of data distribution, the Kolmogorov–Smirnov test was utilized. Descriptive statistics were employed to summarize the data, with categorical variables presented as frequencies and percentages, and quantitative variables reported as either means with standard deviations for normally distributed data or medians with interquartile ranges for non-normally distributed data. A generalized linear model (GLM) analysis was selected to explore the relationships between oral health knowledge and various sociodemographic factors, as well as participants’ self-assessed oral health status and daily oral health practices. The GLM allows for the examination of multiple predictors simultaneously while accommodating the categorical and continuous nature of the variables. This method was chosen for its ability to provide robust statistical inference and adjust for potential confounding variables. The significance level for all statistical tests was set at *p* < 0.05 to determine statistically significant associations. Data analysis was conducted using the Statistical Package for the Social Sciences, version 26 (SPSS, IBM Corp, Armonk, NY, USA).

## 3. Results

### 3.1. Sociodemographic Characteristics

Table 1 presents an overview of the socioeconomic characteristics of the study participants and their association with oral health knowledge. This study involved 246 participants, of whom 80.5% (N = 198) were women and 19.5% (N = 48) were men. The median age of the participants was 26.14 ± 8.81 (Md = 23.00, IQR 19.00–30.00, min = 18, max = 57). On average, the participants wore orthodontic braces for 14.72 ± 11.50 months (Md = 12.00, IQR 7.00–19.00, min = 3, max = 48). The total knowledge score on 10 questions about oral health was 8.52 ± 1.61 (Md = 9.00, IQR 8.00–10.00, min = 2, max = 10), with approximately 35.4% of the participants answering all the questions correctly. Over half of the participants (61.0%) had a level of knowledge at or above the median. Among the sociodemographic characteristics of the respondents, none had an impact on the participants’ knowledge level. The majority of the participants were young adults, with 75.6% aged between 18 and 30 years. Most were students (50.0%), with an average self-assessed socioeconomic status of 85.8%.

### 3.2. Oral Health Knowledge Assessment

**Figure 1:**
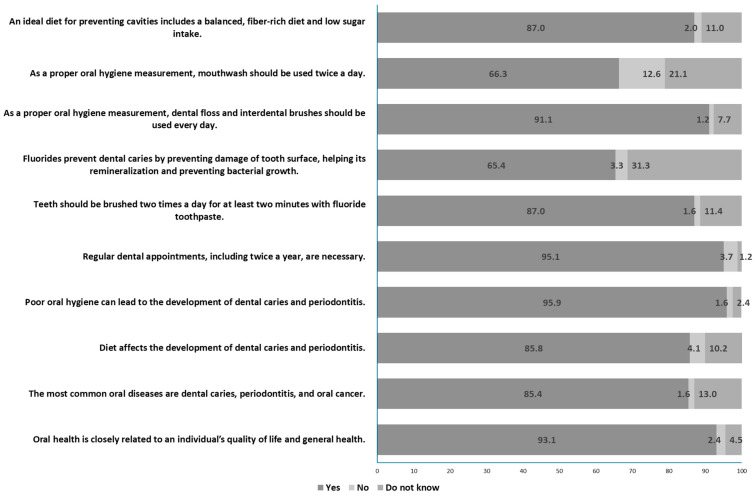
Participants’ responses to oral health knowledge assessment questions.

Figure 1 illustrates the frequencies of correct and incorrect answers to the questions related to oral health and oral hygiene knowledge. Over 80% of the respondents successfully answered 8 out of 10 questions. Almost all of the respondents (N = 236, 95.9%) identified that poor oral hygiene could lead to the development of dental caries and periodontitis. However, the question that received the lowest rate of correct answers was “Fluorides prevent dental caries by preventing damage to the tooth surface, aiding in its remineralization, and inhibiting bacterial growth”, with only 65.4% (N = 161) of the respondents answering accurately.

### 3.3. Self-Reported Oral Health and Hygiene Behavior

Table 2 outlines the questions pertaining to the self-assessment of oral health and oral hygiene knowledge and the duration of orthodontic therapy. The majority of the participants (N = 142, 57.7%) had been receiving orthodontic therapy for up to a year, and 83.3% of them had been previously educated in oral health and oral hygiene. Most of the participants (N = 157, 63.8%) reported difficulties in maintaining oral hygiene due to orthodontic therapy. More than half of them (N = 147, 59.8%) considered oral health and oral hygiene knowledge extremely important. Among the examined variables, only previous education on oral health and oral hygiene had an impact on a higher level of knowledge (β = 0.869, 95% CI 0.263–1.447, *p* = 0.005).

Table 3 outlines the prevalence of self-reported oral conditions in the respondents. The most common issue encountered by the orthodontic patients in this study was the presence of food debris between teeth (N = 114, 46.3%). Tartar was reported by 35.4% of the respondents and 30.9% noted having tooth discolorations. The individuals who experienced teeth sensitivity to cold demonstrated statistically significantly lower oral health knowledge levels (*p* = 0.044).

Table 4 illustrates the frequency of daily oral hygiene aid usage and practices and their association with oral health knowledge. Almost all of the participants (N = 234, 95.1%) used toothbrushes and toothpaste twice or more daily. It was observed that the use of auxiliary oral hygiene aids was relatively low among the respondents. Specifically, 58.9% (N = 145) of the participants reported using interdental brushes, 36.6% (N = 90) used dental floss, and only 27.6% (N = 68) regularly used mouthwash. Slightly more than half of the respondents (N = 136, 55.3%) used a special orthodontic toothbrush, and these individuals demonstrated significantly higher levels of oral health knowledge (*p* = 0.032). Higher oral knowledge level scores have also been connected with the orthodontic patients who use an electric rotary–oscillating toothbrush (*p* ≤ 0.001 *) and visit a dentist once a year (*p* = 0.039).

### 3.4. Reasons for Choosing Orthodontic Therapy and Expectations

Table 5 presents the distribution of reasons for choosing orthodontic therapy among the respondents. The most prevalent reason was to improve aesthetics (N = 157, 63.8%), followed by enhancing oral function (N = 122, 49.6%), and improving self-esteem (N = 105, 42.7%).

**Figure 2:**
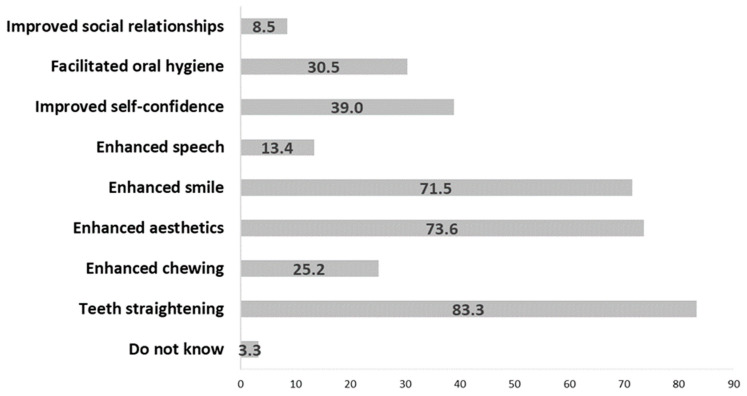
Patient expectations from fixed orthodontic therapy.

Figure 2 provides information on the expectations of orthodontic therapy among the respondents. The primary expectations of orthodontic therapy among the participants were teeth straightening (N = 205, 83.3%), enhanced aesthetics (N = 181, 73.6%), and an improved smile (N = 176, 71.5%).

### 3.5. Challenges Associated with Orthodontic Treatment

Table 6 illustrates the negative characteristics associated with wearing fixed orthodontic appliances. The participants in this study highlighted poor aesthetics (N = 76, 30.9%), the long duration of therapy (N = 68, 27.6%), and the occurrence of mouth ulcers (N = 59, 24.0%) as the most prevalent problems in orthodontic therapy.

## 4. Discussion

This study found that adult orthodontic patients have a high level of knowledge about oral health. This study found no statistically significant differences in the oral health knowledge among the respondents, regardless of gender, age, educational level, employment status, or socioeconomic status. In contrast, a study examining oral health knowledge, attitudes, and practices among orthodontic and non-orthodontic patients in an Indian dental institute found that male orthodontic patients tended to have greater oral health knowledge, while female patients showed a more proactive approach to oral health attitudes and practices. In that study, the orthodontic patients demonstrated significantly higher oral health knowledge compared to the non-orthodontic patients [29]. Similar findings were observed in a multicenter study conducted in France, wherein adolescents undergoing orthodontic treatment exhibited a greater knowledge of oral health compared to adolescents without such treatment [12].

Among the 10 questions used for determining the oral health knowledge, over 80% of the respondents successfully answered 8 out of 10 questions. The participants’ high level of oral health knowledge can be attributed to effective public health campaigns, access to education, and proactive personal health management [12]. During orthodontic therapy, retention sites like orthodontic bands, brackets, wires, and acrylic resins have the potential to accumulate bacterial plaque. Prolonged plaque accumulation in these areas can contribute to the development of tooth decay and periodontal disease [30,31]. Nearly all (95.9%) of the orthodontic patients in this study demonstrated an awareness that inadequate oral hygiene could contribute to the onset of dental caries and periodontitis. In a study conducted in Jordan, orthodontic patients undergoing fixed or removable appliance therapy exhibited limited periodontal knowledge. Specifically, 95% of the participants were unable to recognize the consequences of inadequate oral hygiene and dental plaque buildup [22]. Fluorides play a crucial role in orthodontic therapy. The use of fluoridated mouthrinses significantly decreases enamel decalcification and reduces gingival inflammation throughout orthodontic treatment. It is advised that individuals with fixed orthodontic appliances incorporate daily rinsing with a 0.05% sodium fluoride mouthwash as part of their oral hygiene regimen [32]. In contrast, only 66.3% of the orthodontic patients in this study acknowledged the significance of incorporating mouthwashes into their daily oral hygiene routine, and a mere 65.4% were aware of how fluorides prevent tooth decay and strengthen enamel. Similar deficiencies in fluoride awareness were identified in a French study involving adolescents undergoing fixed orthodontic treatment [12].

Research findings consistently indicate that oral health education is instrumental in improving knowledge, attitudes, and practices concerning oral hygiene. Moreover, such education has been shown to decrease plaque accumulation, bleeding on probing of the gingival tissue, and the development of new cavities, while concurrently fostering better gingival health [33]. In the course of this study, it was observed that the orthodontic patients who had undergone prior education sessions focusing on oral health and hygiene exhibited notably elevated levels of oral health knowledge. This finding underscores the positive impact of educational interventions on patients’ understanding of oral health concepts and practices. Using interdental brushes, floss threaders, and specialized toothbrushes, along with maintaining a consistent cleaning routine and avoiding sticky foods, can greatly improve oral hygiene during orthodontic treatment. Inadequate oral hygiene practices during orthodontic treatment could jeopardize the completion of treatment, as the increased risk of tissue damage, such as tooth demineralization and periodontal inflammation, may necessitate the premature termination of therapy [27]. A majority of orthodontic patients (63.8%) participating in this study expressed challenges in maintaining oral hygiene as a result of their ongoing orthodontic treatment. This issue was also identified in a Brazilian study involving orthodontic patients, where 53.3% reported difficulties in flossing their teeth, and 16.7% encountered challenges with brushing due to their orthodontic therapy [27]. Adolescents undergoing orthodontic therapy in a study from Lithuania also reported changes in their oral hygiene habits, with 36.4% of them indicating that their oral hygiene routine changed significantly due to the treatment [26]. Most of the orthodontic patients in this study rated their oral health, oral hygiene, and their knowledge of oral health and hygiene as very good or excellent. Similar findings were reported in a study conducted among orthodontic clients from China and New Zealand. In this study, 79.4% of the participants rated their oral hygiene as good, while only 24.3% assessed their oral health knowledge as good [9].

The most prevalent self-reported issue affecting the current oral health status among orthodontic patients was the accumulation of food debris between teeth, reported by 46.3% of the participants. This issue was similarly reported by 31.3% of the orthodontic patients undergoing fixed or removable appliance therapy in a Saudi Arabian study [21]. Diet plays a crucial role in oral health, particularly for orthodontic patients, as the type of food trapped between brackets influences the nature of plaque buildup. Increased sugar intake can elevate the risk of developing caries and white spot lesions. Moreover, sticky foods are more prone to remaining lodged between brackets for extended periods. Consequently, orthodontists advise patients to avoid sticky foods and reduce their sugar consumption [34]. Other self-reported oral health issues included tartar (35.4%), tooth discoloration (30.9%), and tooth sensitivity to cold (26.4%). Only 19.1% of the orthodontic patients in this study reported struggling with bleeding gums, which is significantly lower compared to the 51.0% of orthodontic patients in China and New Zealand who reported experiencing bleeding gums while brushing [9].

During treatment with fixed appliances, maintaining proper oral hygiene is essential. This involves brushing teeth twice daily with fluoride toothpaste. Additionally, orthodontic patients are encouraged to perform interdental cleaning at least once a day. As a complement to these practices, using mouthwash twice a day is recommended [28]. Various ingredients found in mouthwashes prevent oral issues such as decay, gingivitis, tartar, bad breath, mouth ulcers, and dentin sensitivity [13]. The majority of the orthodontic patients in this study demonstrated favorable oral hygiene practices, with 95.1% of them reporting using a toothbrush and toothpaste at least twice a day or more. These findings align with previous studies on periodontal health knowledge and awareness among subjects with fixed orthodontic appliances [22] and the oral hygiene habits and status of orthodontic patients attending the University of Pretoria, Oral and Dental Hospital [24]. Conversely, other studies from China and New Zealand [9], Pakistan [25], Lithuania [26], and India [28,29] reported that less than 80% of orthodontic patients exhibited these oral hygiene habits. Studies suggest that orthodontic toothbrushes are more effective than conventional toothbrushes in maintaining oral health among fixed orthodontic patients. The patients who utilized orthodontic toothbrushes showed an improved oral and dental health status, healthier gingiva, and lower rates of caries compared to those using conventional toothbrushes [35]. In this study, slightly more than half (55.3%) of the orthodontic patients incorporated special orthodontic toothbrushes into their daily oral hygiene routine. Interestingly, these individuals also exhibited significantly higher levels of oral health knowledge. The orthodontic patients from this study mostly used toothbrushes with medium bristles (52.0%). This type of toothbrush was also preferred by 44.0% of orthodontic patients from Pakistan [25] and 60.71% from India [28]. The usage of oral mouthwash as part of an oral hygiene routine in this study was low, with only 27.6% of the participants reporting regular use. This is especially important as oral mouthwashes have demonstrated effectiveness in managing cariogenic plaque in patients with fixed orthodontic appliances [36]. Studies assessing oral hygiene habits among orthodontic patients reveal diverse findings on this matter. The use of mouthwash was significantly higher in studies among patients receiving orthodontic treatment in Jordan [22], Malta [23], at the University of Pretoria’s Oral and Dental Hospital [24], in China and New Zealand [9], in Pakistan [25], and at a dental institute in India [29]. On the other hand, only 23.88% of patients visiting dental clinics in Patna, according to an Indian study, used mouthwash in their oral hygiene routine [28]. Orthodontic patients in this study have also demonstrated limited usage of dental floss, with only 36.6% incorporating it into their routine regularly. The usage of dental floss has also been shown to be even lower, with less than 10% of orthodontic patients from studies conducted in China and New Zealand [9], Pakistan [25], dental clinics in Patna [28], and India [29] reporting its use. Conversely, more than half (56.0%) of orthodontic patients attending the University of Pretoria’s Oral and Dental Hospital reported regular usage of dental floss [24]. The usage of interdental brushes was the most prominent among other auxiliary aids. Over half (58.9%) of the participants in this study reported employing interdental brushes daily. These findings were similar in studies among orthodontic patients from Jordan [22], the University of Pretoria’s Oral and Dental Hospital [24], Pakistan [25], France [12], and India [29], where more than 50% of their participants incorporated interdental brushes into their regular oral hygiene routine. Nevertheless, certain studies indicated a remarkably low utilization of interdental brushes, with figures such as 15.3% among orthodontic patients in China and New Zealand [9], and a mere 0.74% among patients visiting dental clinics in Patna [28]. It is worth noting that in this study, a significant portion of orthodontic patients (79.3%) brushed their teeth for 2 to 3 min, while 58.1% replaced their toothbrush within a 3-month period. Other studies have yielded varied findings. A study from Lithuania among adolescents receiving orthodontic treatment showed that 81.8% of their participants brushed their teeth for 2 to 3 min [26]. On the other hand, studies from China and New Zealand [9] and France [12] revealed shorter durations of toothbrushing among their orthodontic patients. Approximately half of the orthodontic patients in studies from Malta [23] and China and New Zealand [9] replaced their toothbrushes within 3 months. However, a study among patients visiting dental clinics in Patna revealed that 77.67% of their participants replaced their toothbrushes within one month [28].

Adults pursue orthodontic treatment for a multitude of reasons, including enhancing aesthetics, addressing functional needs like bite corrections, boosting self-confidence, and enhancing oral hygiene [1,2,3]. In this study, aesthetic improvement played a significant role in the decision-making process for this treatment for 63.8% of the respondents, followed by enhancing occlusion, which was cited by 49.6% of the participants. In the findings from a study on the expectations of orthodontic treatments among adults in Brazil, it was revealed that occlusion deviation stood out as the primary motivation for seeking treatment, noted by 66.7% of the participants. Following closely, aesthetics emerged as the second most significant factor, with 48.3% highlighting its importance [27].

Orthodontic therapy is often associated with several negative characteristics, including discomfort, dietary restrictions, oral hygiene challenges, and social interactions [5,6,7,8]. In this study, the orthodontic patients highlighted poor aesthetics (30.9%), the lengthy duration of therapy (27.6%), and mouth ulcers (24.0%) as the most prevalent negative issues in orthodontic treatment. On the other hand, a study on the expectations of orthodontic treatments among adults in Brazil identified pain (58.3%) as the main complaint about treatment [27].

This study’s findings must be interpreted within the context of its numerous limitations. This study was constrained by its modest sample size, drawn exclusively from a single private specialist orthodontic practice. Consequently, the findings may not be readily generalizable to a wider population. It would be advantageous to undertake a study with a larger participant pool to enhance the external validity of the results. Additionally, this study employed convenience sampling for participant recruitment, which can introduce selection bias and limit the ability to control for confounding variables effectively. Furthermore, this study acknowledged the potential for social desirability bias, as participants may have provided responses they perceived as socially acceptable, potentially affecting the accuracy of the data. Moreover, due to the time constraints of this study, there may have been limitations in capturing the long-term changes in the oral health behaviors or outcomes. Lastly, this study’s geographical and demographic specificity, limited to a single orthodontic practice, may restrict the generalizability of the findings to broader populations or different geographic regions. Therefore, future research should aim for larger, more diverse samples across various settings to strengthen the applicability and robustness of the findings. Employing randomized sampling methods and longitudinal study designs could further mitigate biases and provide more comprehensive insights into oral health education and practice improvements.

Based on the findings of this study, particularly in relation to oral health education and practice improvement, specific recommendations for future research include the implementation of targeted educational interventions tailored to improve oral health knowledge among different demographic groups [37]. It is also recommended to investigate the effectiveness of community-based oral health programmes aimed at improving oral hygiene practices. In addition, future research should investigate the long-term effects of orthodontic therapy on oral health and patient satisfaction. Given these limitations, it is crucial to emphasise the need for targeted interventions aimed at improving oral health education and promoting effective oral hygiene practices among orthodontic patients. Educational programmes should focus on addressing the specific gaps identified in this study, such as the low use of aids such as floss and mouthwash. By improving oral health knowledge and promoting better oral hygiene practices, such interventions have the potential to significantly reduce dental complications during orthodontic treatment and improve patients’ overall oral health.

## 5. Conclusions

This study sheds light on the motivation and oral health practices of patients undergoing fixed orthodontic therapy. While the participants demonstrated a commendable level of oral health knowledge, there is room for improvement in their oral hygiene habits, particularly in the use of tools such as interdental brushes, floss, and mouthwash, as well as chemical compounds in oral hygiene products to improve oral health. In this study’s cohort, the improvement of aesthetics in particular proved to be the main reason for seeking orthodontic treatment. It is therefore recommended that educational programmes for adult patients considering fixed orthodontic treatment should emphasise the importance of incorporating additional appliances into their oral hygiene routines. In addition, regular dental visits are crucial not only for maintaining oral health, but also for monitoring treatment progress and treating any complications that arise. By implementing these recommendations, clinicians can better support their patients in achieving optimal oral health outcomes throughout their orthodontic treatment. Ongoing education and support for orthodontic patients is essential to ensure they are using interdental brushes, floss, and specialized toothbrushes effectively, maintaining a consistent brushing routine and avoiding sticky foods to improve their oral and overall health during treatment.

## Figures and Tables

**Table 1:** Sociodemographic predictors of enhanced oral health knowledge.

Characteristic		N (%)	Knowledge Score	
			*β* (95% CI)	*p*-Values
Gender	Woman	198 (80.5)	Reference	
	Man	48 (19.5)	0.462 (−0.284–1.209)	0.225
Age group (years)	18–30	186 (75.6)	Reference	
	31–45	50 (20.3)	0.119 (−0.693–0.931)	0.774
	>45	10 (4.1)	0.621 (−0.911–2.153)	0.427
Education level	High school	172 (69.9)	Reference	
	Bachelor’s degree	33 (13.4)	−0.322 (−1.180–0.535)	0.461
	Master’s degree	34 (13.8)	−0.469 (−1.305–0.367)	0.271
	MSc or PhD	7 (2.8)	−1.178 (−3.064–0.708)	0.221
Employment status	Student	123 (50.0)	Reference	
	Employed	107 (43.5)	0.100 (−0.597–0.796)	0.779
	Unemployed	16 (6.5)	−0.006 (−1.172–1.160)	0.992
Socioeconomic status	Below average	9 (3.7)	Reference	
	Average	211 (85.8)	0.033 (−1.394–1.460)	0.963
	Above average	26 (10.6)	−0.154 (−1.827–1.518)	0.856

Data are presented as numbers (percentages). Reference knowledge category is “low”. *p* ≤ 0.05. Abbreviations: *β*, regression coefficients; 95% CI, 95% confidence interval.

**Table 2:** Self-reported oral health and oral hygiene behavior.

Characteristic		N (%)
Duration of orthodontic therapy (months)	3–12	142 (57.7)
	13–24	66 (26.8)
	>24	38 (15.4)
Impact of orthodontic treatment on oral hygiene maintenance	Difficulties	157 (63.8)
	No difficulties	89 (36.2)
Educated on the topic of oral health and oral hygiene	Yes	205 (83.3)
	No	41 (16.7)
Interested in further education on the topic of oral health and oral hygiene	Yes	156 (63.4)
	No	90 (36.6)
Importance of oral health and oral hygiene knowledge	Extremely important	147 (59.8)
	Very important	89 (36.2)
	Moderately important	10 (4.1)
Source of oral health and oral hygiene information *	Family	132 (53.2)
	Dentist	209 (84.3)
	Media	106 (42.7)
	School	72 (29.0)
	Other	5 (2.0)
Self-assessment of oral health knowledge	Excellent	49 (19.9)
	Very good	111 (45.1)
	Moderate	80 (32.5)
	Poor	6 (2.4)
Self-assessment of oral hygiene knowledge	Excellent	62 (25.2)
	Very good	120 (48.8)
	Moderate	57 (23.2)
	Poor	7 (2.8)
Self-assessment of oral health status	Excellent	62 (25.2)
	Very good	112 (45.5)
	Moderate	64 (26.0)
	Poor	8 (3.3)
Self-assessment of oral hygiene	Excellent	74 (30.1)
	Very good	108 (43.9)
	Moderate	62 (25.2)
	Poor	2 (0.8)

Data are presented as numbers (percentages). * Multiple answers possible.

**Table 3:** Correlation between self-reported oral health status and oral health knowledge.

Oral Health Status	N (%)	Knowledge Score	
		*β* (95% CI)	*p*-Values
Plaque	35 (14.2)	−0.120 (−0.967–0.728)	0.782
Tartar	87 (35.4)	−0.192 (−0.806–0.422)	0.541
Tooth discoloration	76 (30.9)	0.440 (−0.181–1.061)	0.165
Gum inflammation	19 (7.7)	−0.087 (−1.106–0.931)	0.867
Gum bleeding	47 (19.1)	−0.184 (−0.887–0.519)	0.608
Bad breath	20 (8.1)	0.029 (−0.993–1.051)	0.955
Dry mouth	22 (8.9)	−0.001 (−0.971–0.968)	0.998
Burning mouth	7 (2.8)	−0.363 (−2.005–1.278)	0.665
Food debris between teeth	114 (46.3)	−0.393 (−0.959–0.174)	0.174
Tooth sensitivity to temperature changes	54 (22.0)	0.394 (−0.684–1.471)	0.474
Tooth sensitivity to cold	65 (26.4)	−1.043 (−2.057–0.029)	0.044 *

Data are presented as numbers (percentages). Reference knowledge category is “low” and “answer no”. * *p* ≤ 0.05. Abbreviations: *β*, regression coefficients; 95% CI, 95% confidence interval.

**Table 4:** The relationship between self-reported frequency of oral hygiene aid usage, oral health behavior, and oral health knowledge.

Characteristic		N (%)	Knowledge Score	
			*β* (95% CI)	*p*-Values
Toothbrush	Regular (≥2× day)	234 (95.1)	21.641 (−89,678.056–89,721.337)	1.000
	Irregular (<2× day)	12 (4.9)	Reference	
Dental floss	Regular (daily)	90 (36.6)	0.562 (−0.130–1.255)	0.112
	Irregular	156 (63.4)	Reference	
Interdental brush	Regular (daily)	145 (58.9)	−0.171 (−0.843–0.501)	0.618
	Irregular	101 (41.1)	Reference	
Mouthwash	Regular (daily)	68 (27.6)	0.293 (−0.427–1.012)	0.425
	Irregular	178 (72.4)	Reference	
Toothpaste	Regular (≥2× day)	234 (95.1)	−23.630 (−89,723.327–89,676.066)	1.000
	Irregular (<2× day)	12 (4.9)	Reference	
Tongue scraper	Regular (daily)	48 (19.5)	0.987 (0.099–1.876)	0.029 *
	Irregular	198 (80.5)	Reference	
Usage of special orthodontic toothbrush	Yes	136 (55.3)	0.674 (0.058–1.290)	0.032 *
	No	110 (44.7)	Reference	
Hardness of toothbrush	Soft	89 (36.2)	0.102 (−0.942–1.145)	0.848
	Ultrasoft	25 (10.2)	Reference	
	Medium	128 (52.0)	0.492 (−0.532–1.516)	0.346
	Hard	4 (1.6)	−2.165 (−5.179–0.850)	0.159
Frequency of toothbrush replacement	Every month	74 (30.1)	0.043 (−1.076–1.161)	0.941
	Every three months	143 (58.1)	0.221 (−0.766–1.208)	0.661
	More than three months	29 (11.8)	Reference	
Type of toothbrush used	Manual	169 (68.7)	Reference	
	Electric rotary–oscillating	54 (22.0)	1.630 (0.753–2.507)	≤0.001 *
	Electric sonic vibrating	23 (9.3)	0.426 (−0.625–1.477)	0.427
Duration of brushing	<2 min	18 (7.3)	Reference	
	2–3 min	195 (79.3)	−0.934 (−2.169–0.301)	0.138
	>3 min	33 (13.4)	−0.800 (−2.245–0.646)	0.278
Frequency of dental visits	Every 6 months	95 (38.6)	0.102 (−0.550–0.754)	0.759
	Once a year	55 (22.4)	0.889 (0.044–1.734)	0.039 *
	As needed	96 (39.0)	Reference	

Data are presented as numbers (percentages). Reference knowledge category is “low”. * *p* ≤ 0.05. Abbreviations: *β*, regression coefficients; 95% CI, 95% confidence interval.

**Table 5:** Reasons that influenced the decision for orthodontic therapy among participants.

Characteristics	Level of Influence N (%)				
	Extremely Influential	Very Influential	Somewhat Influential	Slightly Influential	Not at All Influential
Improvement of oral health	75 (30.5)	63 (25.6)	71 (28.9)	15 (6.1)	22 (8.9)
Enhancement of function (bite alignment, occlusion)	122 (49.6)	35 (14.2)	44 (17.9)	15 (6.1)	30 (12.2)
Speech improvement	38 (15.4)	18 (7.3)	57 (23.2)	45 (18.3)	88 (35.8)
Crowding and spacing of teeth	97 (39.4)	35 (14.2)	45 (18.3)	22 (8.9)	47 (19.1)
Increased self-confidence	105 (42.7)	32 (13.0)	51 (20.7)	20 (8.1)	38 (15.4)
Social reasons	37 (15.0)	33 (13.4)	65 (26.4)	34 (13.8)	77 (31.3)
Prosthodontic indications	30 (12.2)	27 (11.0)	56 (22.8)	31 (12.6)	102 (41.5)
TMJ pain	9 (3.7)	11 (4.5)	41 (16.7)	40 (16.3)	145 (58.9)
Oral hygiene maintained	72 (29.3)	48 (19.5)	54 (22.0)	20 (8.1)	52 (21.1)
Aesthetic enhancements	157 (63.8)	38 (15.4)	30 (12.2)	10 (4.1)	11 (4.5)

Data are presented as numbers (percentages).

**Table 6:** Level of agreement with negative characteristics associated with wearing fixed orthodontic appliances.

Characteristic	Level of Agreement N (%)				
	Strongly Agree	Agree	Neither Agree or Disagree	Disagree	Strongly Disagree
Pain	22 (8.9)	56 (22.8)	92 (37.4)	34 (13.8)	42 (17.1)
Duration	68 (27.6)	53 (21.5)	62 (25.2)	32 (13.0)	31 (12.6)
Maintaining oral hygiene	51 (20.7)	57 (23.2)	58 (23.6)	36 (14.6)	44 (17.9)
Costs	59 (24.0)	45 (18.3)	75 (30.5)	23 (9.3)	44 (17.9)
Aesthetics	76 (30.9)	35 (14.2)	60 (24.4)	36 (14.6)	39 (15.9)
Chewing difficulty	35 (14.2)	64 (26.0)	58 (23.6)	40 (16.3)	49 (19.9)
Dietary issues	30 (12.2)	32 (13.0)	63 (25.6)	54 (22.0)	67 (27.2)
Speech difficulty	11 (4.5)	26 (10.6)	58 (23.6)	41 (16.7)	110 (44.7)
Gum swelling	16 (6.5)	33 (13.4)	54 (22.0)	50 (20.3)	93 (37.8)
Gum bleeding	17 (6.9)	25 (10.6)	50 (20.3)	60 (24.4)	94 (38.2)
Gum recession	18 (7.3)	17 (6.9)	51 (20.7)	57 (23.2)	103 (41.9)
Mouth ulcers	59 (24.0)	51 (20.7)	42 (17.1)	37 (15.0)	57 (23.2)
Tooth decay	4 (1.6)	15 (6.1)	53 (21.5)	52 (21.1)	122 (49.6)
TMJ pain	5 (2.0)	12 (4.9)	49 (19.9)	49 (19.9)	131 (53.3)
Headache	6 (2.4)	19 (7.7)	45 (18.3)	48 (19.5)	128 (52.0)
Teeth mobility	8 (3.3)	27 (11.0)	68 (27.6)	41 (16.7)	102 (41.5)
Teeth discoloration	11 (4.5)	18 (7.3)	53 (21.5)	45 (18.3)	119 (48.4)
Socialization difficulties	9 (3.7)	13 (5.3)	39 (15.9)	42 (17.1)	143 (58.1)
Smiling difficulties	30 (12.2)	22 (8.9)	39 (15.9)	45 (18.3)	110 (44.7)

Data are presented as numbers (percentages).

## Data Availability

Data supporting the findings of this study are available upon request from the corresponding author.
